# Supplementary material for: Molecular dissection of box jellyfish venom cytotoxicity highlights an effective venom antidote
Source: Nat Commun. 2019 Apr 30;10:1655. doi: 10.1038/s41467-019-09681-1 (PMC6491561; doi:10.1038/s41467-019-09681-1)
Supplement: Supplementary file 1 — Supplementary Information [file 41467_2019_9681_MOESM1_ESM.pdf]

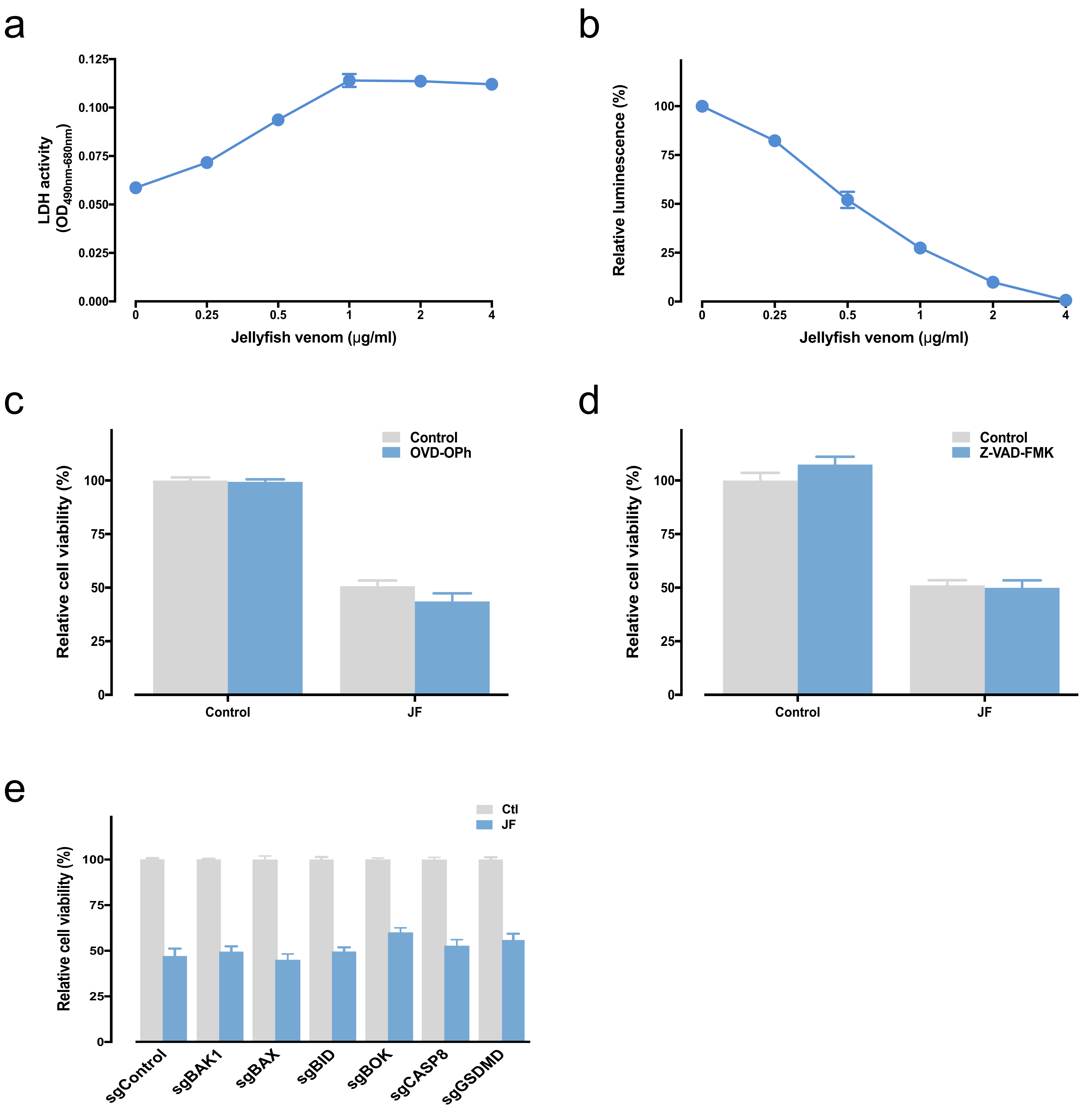

Supplementary Figure 1. Inhibition of apoptotic or pyroptotic pathway alone did not protect cells from venom cytotoxicity. a, b HAP1 cells were treated with the indicated concentrations of jellyfish venom for 24 hrs, and the viability of the cells was determined by (**a**) LDH release assay and (**b**) ATP depletion assay (n=3). **c, d** Caspase inhibition by (**c**) OVD-OPh (10µM) or (**d**) Z-VAD-FMK (25µM) has no effect on the box jellyfish venom-induced cell cytotoxicity (0.75µg/ml; n=3). **e** Depletion of BAK1, BAX, BID, BOK, CASP8 or GSDMD does not confer resistance to jellyfish venom (0.75µg/ml) in HAP1 cells (n=3). Data represented as mean ± S.E.M.

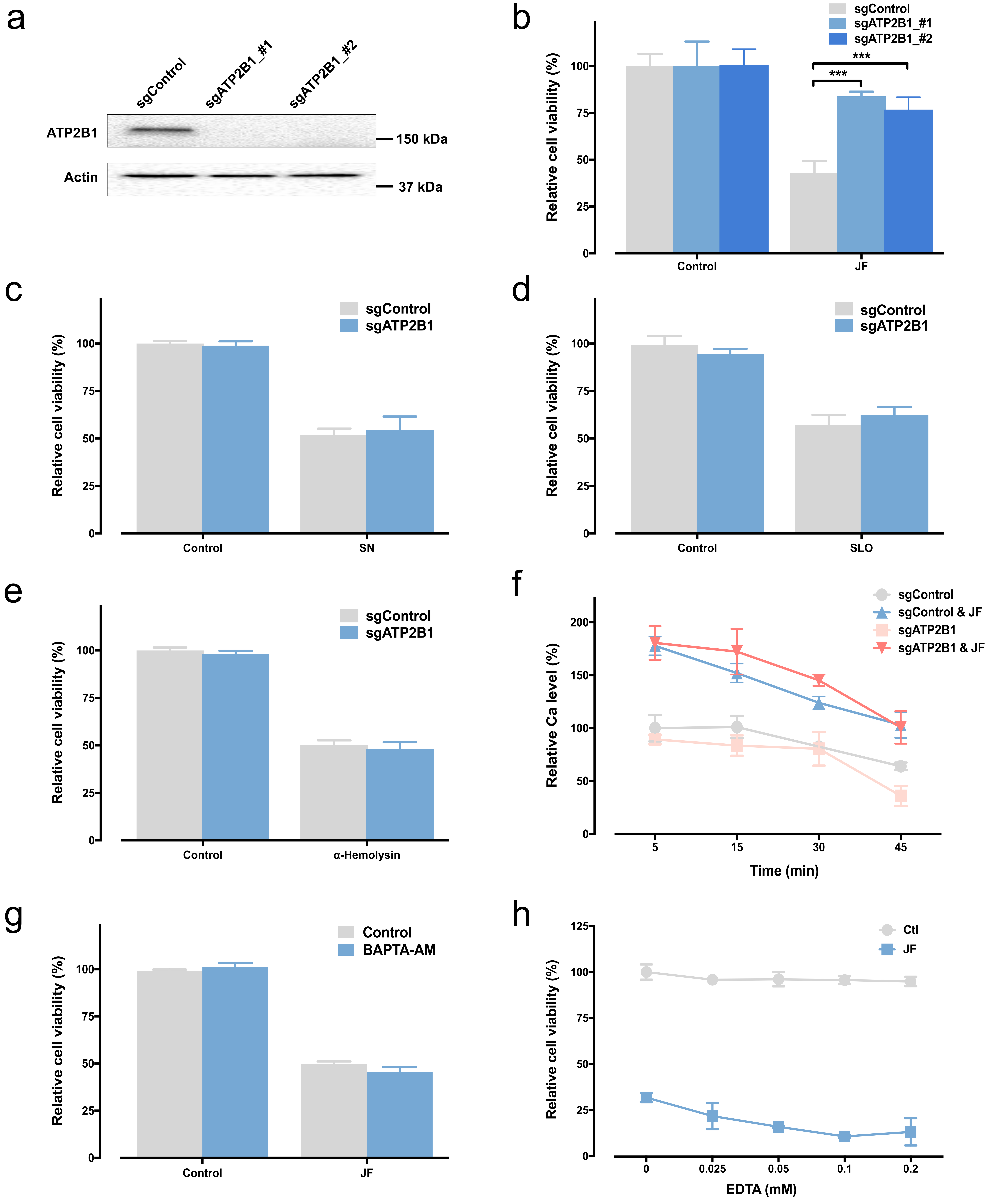

Supplementary Figure 2. Depletion of ATP2B1 conferring resistance to jellyfish venom via calcium-independent pathway. a Western blot validation of clonal ATP2B1 knockout in HAP1 cells. **b** Clonal ATP2B1 knockout cells conferring resistance to box jellyfish venom (0.75μg/ml) in HAP1 cells (n=3). One-way ANOVA followed by Tukey's *post hoc* test, ***, $p < 0.001$. **c-e** Depletion of ATP2B1 does not confer resistance to (**c**) sea nettle (*Chrysaora quinquecirrha*) venom (SN; 4μg/ml)) and pore-forming toxin (**d**) streptolysin O (SLO; 1000U) from *Streptococcus pyogenes* and (**e**) α-hemolysin (0.8μg/ml) from *Staphylococcus aureus* (n=3). **f** The depletion of ATP2B1 produces minimal changes in calcium influx in HAP1 cells after box jellyfish venom treatment. Assay for calcium influx in control (sgControl) and ATP2B1 depletion cells (sgATP2B1) after box jellyfish venom (JF) (n=3). **g, h** Box jellyfish venom induced cell death is calcium-independent. Pre-treatment of (**g**) intracellular calcium chelator, BAPTA-AM, or (**h**) extracellular calcium chelator, EDTA, has no effect on the jellyfish venom-induced cell death (n=3). Data represented as mean ± S.E.M. Significance was assessed using one-way ANOVA followed by Tukey's *post hoc* test, ***, $p < 0.001$.

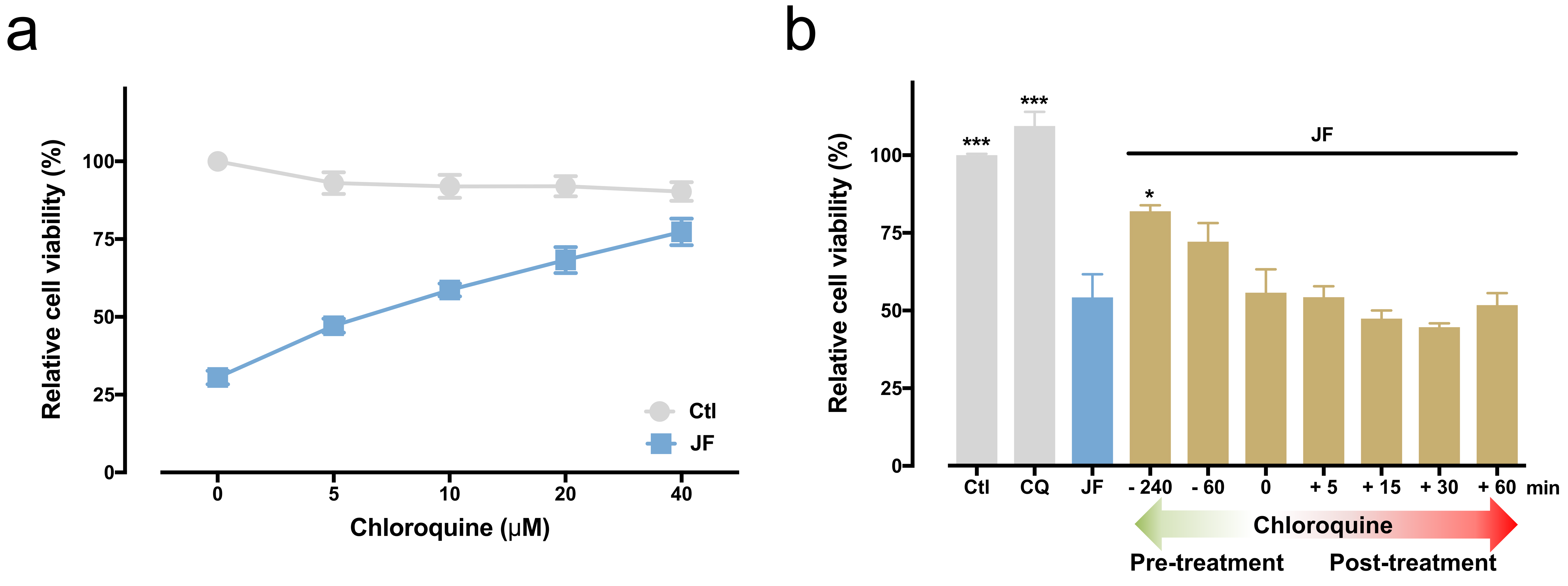

Supplementary Figure 3. Effects of chloroquine on jellyfish venom-induced cell death. a HAP1 cells were pre-treated with the indicated concentration of chloroquine (CQ) for 4hrs and treated with jellyfish venom (1μg/ml) for 24hrs (n=3). **b** Comparison of pre-treatment and post-treatment of chloroquine in protection of the HAP1 cells from jellyfish venom (0.75μg/ml; n=3). All data represented as mean ± S.E.M. One-way ANOVA followed by Tukey's *post hoc* test, ***,** p <0.05; ***, p <0.001.

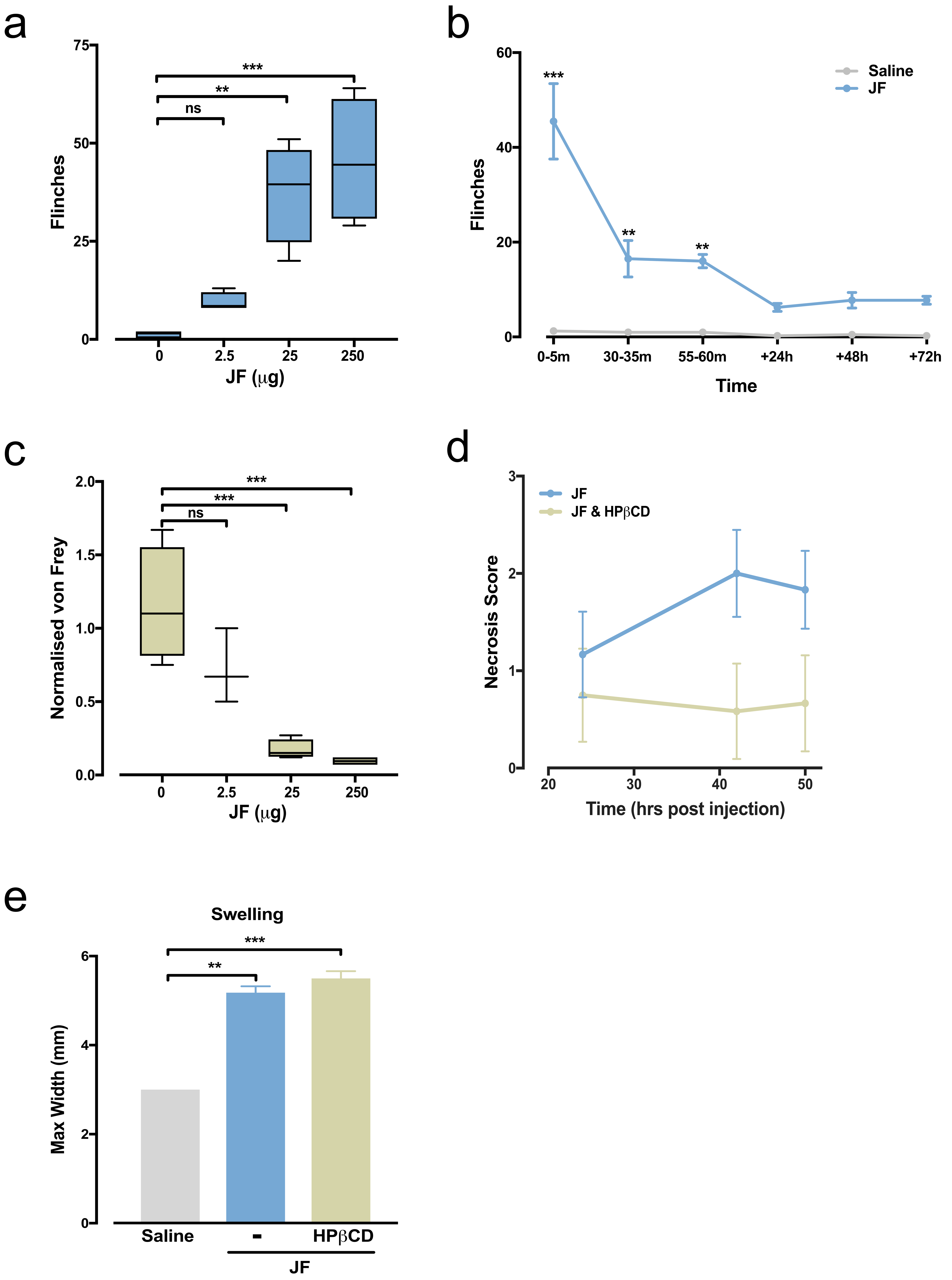

Supplementary Figure 4. Effects of jellyfish venom *in vivo.* **a** Box plots showing pain response from flinching behaviour on mice treated with intraplantar injection of various dose of jellyfish venom alone (n=4; center line, median; box limits, upper and lower quartiles; whiskers, max to min). **b** time course of pain response from flinching behaviour on mice treated with intraplantar injection of jellyfish venom alone. **c** Box plots showing pain response from von Frey test for mechanical allodynia on mice treated with intraplantar injection of various dose of jellyfish venom alone (n=4; center line, median; box limits, upper and lower quartiles; whiskers, max to min). **d** time course of pain response from flinching behaviour on mice treated with intraplantar injection of jellyfish venom alone or co-injection with HPβCD. **e** Swelling effects after injection of jellyfish venom alone or co-injection with HPβCD (n=7-16). All data represented as mean ± S.E.M. (**a**, **c**, and **e**) One-way ANOVA followed by Tukey's *post hoc* test, ***,** p <0.05; **, p <0.01; ***, p <0.001; ns, not significant. (b) Two-way ANOVA followed by Bonferroni's multiple comparisons test, **, p <0.01; ***, p <0.001.

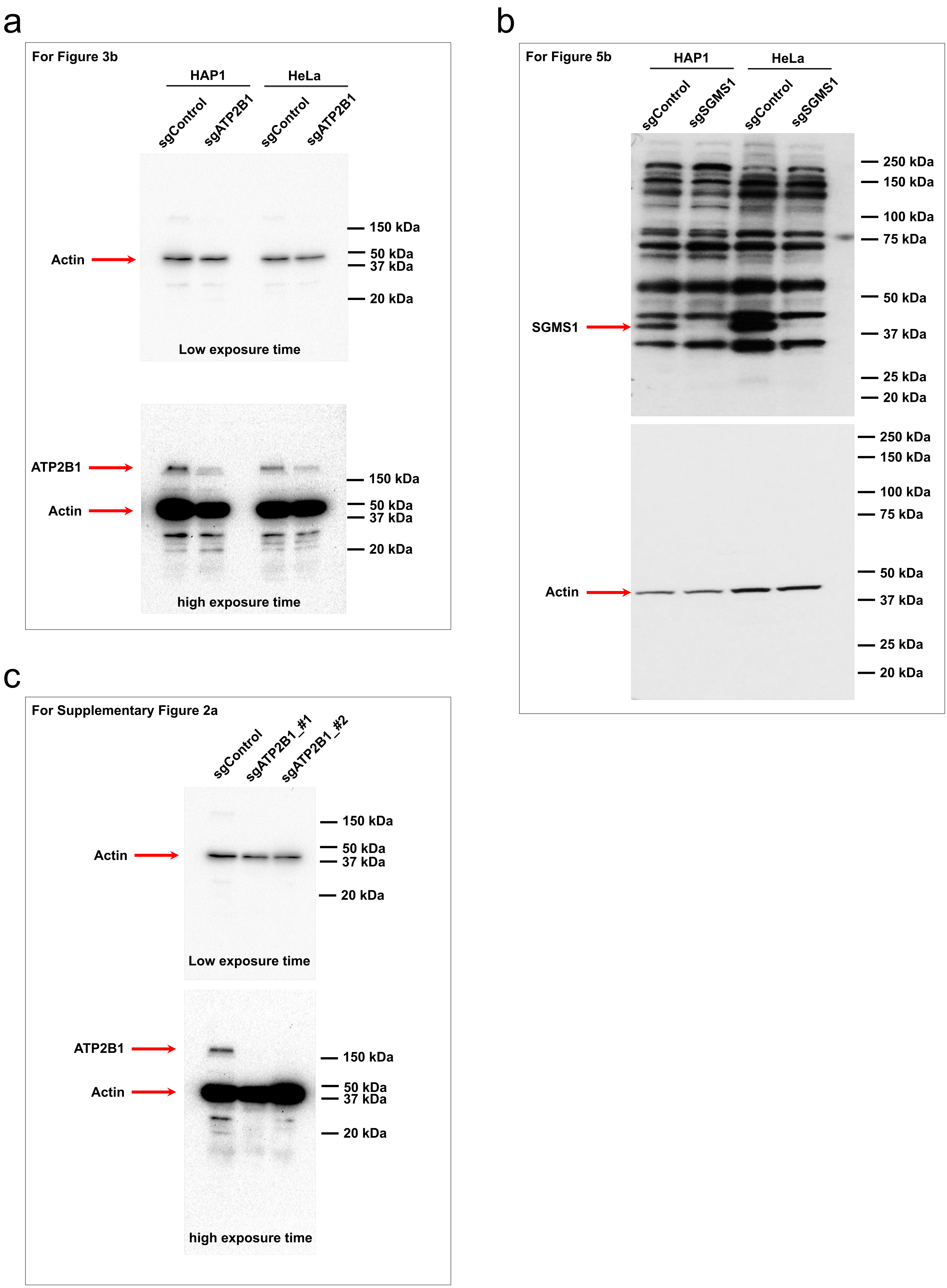

Supplementary Figure 5. Uncropped images of Western blot (**a**) for Fig. 3b, (**b**) for Fig. 5b and (**c**) for Supplementary Fig. 2a.

Supplementary Table1. This table contains enriched GO terms and pathways of the top hits in screen.

p-value	q-value	pathway	source	external_id	members_input_overlap	members_input_overlap_geneids	effective_size	log10
9.43E-05	0.00886209	Regulation of cholesterol biosynthesis by SREBP (SREBF)	Reactome	R-HSA-1655829	SREBF2; SCAP; MBTPS2; MBTPS1	6721; 51360; 22937; 8720	31	4.02559146
0.00035981	0.01691107	ATF6 (ATF6-alpha) activates chaperones	Reactome	R-HSA-381033	MBTPS2; MBTPS1	8720; 51360	4	3.44392671
0.003182391	0.09971491	Apoptotic cleavage of cell adhesion proteins	Reactome	R-HSA-351906	OCLN; DSG3	1830; 100506658	11	2.4972465
0.005952974	0.1398949	Unfolded Protein Response (UPR)	Reactome	R-HSA-381119	MBTPS2; MBTPS1	51360; 8720	15	2.22526599
0.009499831	0.17859683	Other semaphorin interactions	Reactome	R-HSA-416700	PTPRC; PLXNA4	91584; 5788	19	2.02228411
0.02144257	0.3359336	Endosomal Sorting Complex Required For Transport (ESCRT)	Reactome	R-HSA-917729	STAM2; VPS25	84313; 10254	29	1.66872316
0.025802869	0.33764437	Amino acid synthesis and interconversion (transamination)	Reactome	R-HSA-70614	GLS; ASNS	2744; 440	32	1.58833201
0.033786781	0.33764437	Metalloprotease DUBs	Reactome	R-HSA-5689901	HIST1H2AH; HIST1H2AJ	85235; 8331	37	1.47125318
0.03721644	0.33764437	Apoptotic cleavage of cellular proteins	Reactome	R-HSA-111465	OCLN; DSG3	100506658; 1830	39	1.42926517
0.037331258	0.33764437	Regulation of TP53 Activity through Phosphorylation	Reactome	R-HSA-6804756	PRKAG2; TAF3; CSNK2A2	83860; 51422; 1459	94	1.42792737
0.046338479	0.33764437	Signaling by Retinoic Acid	Reactome	R-HSA-5362517	PDHB; RDH5	5959; 5162	44	1.33405823
0.046338479	0.33764437	Sphingolipid de novo biosynthesis	Reactome	R-HSA-1660661	FA2H; SGMS1	259230; 79152	44	1.33405823

Supplementary Table 2. sgRNA sequences used for gene validation.

Gene ID	sgRNA
MLKL	ATCCCCGTGGATTCTGCTAA
ATP2B1	AGAAAGGCCCAATGATACTA
SGMS1	AGCGCATGACCACTACACTG
SCAP	GTGGACTCTGACCGCAAACA
MBTPS1	CACCTGCCACGAATGTGCCA
MBTPS2	GCTCATCACTGAAGTTGCTG
BAK1	GGACGGCAGCTCGCCATCAT
BAX	AGCGAGTGTCTCAAGCGCAT
BID	CCTCACTCACCTCCTCCGAC
BOK	TGTGGTGACCGATGCGTTCC
CAPS8	GTCATCATCCAGTTTGCATT
GSDMD	TGGCCAGGCGCCTTCGCTCG
Control	CCATATCGGGGCGAGACATG

Supplementary Data 1. This table shows frequency of sgRNA deep sequencing reads. Data was obtained by deep-sequencing sgRNA from unselected or surviving jellyfish venome-selected cells. Results are sorted by the top fold-enrichment of an individual sgRNA from the venom-selected cells compared to unselected cells.

Supplementary Data 2. This table contains a list of gene hits and scores after MAGeCK analysis.
