# Supplementary material for: Molecular dissection of box jellyfish venom cytotoxicity highlights an effective venom antidote
Source: Nat Commun. 2019 Apr 30;10:1655. doi: 10.1038/s41467-019-09681-1 (PMC6491561; doi:10.1038/s41467-019-09681-1)
Supplement: Supplementary file 5 — Reporting Summary [file 41467_2019_9681_MOESM5_ESM.pdf]

nature research

Corresponding author(s): G. Gregory Neely

Last updated by author(s): Mar 15, 2019

Reporting Summary

Nature Research wishes to improve the reproducibility of the work that we publish. This form provides structure for consistency and transparency in reporting. For further information on Nature Research policies, see Authors & Referees and the Editorial Policy Checklist.

Statistics

For all statistical analyses, confirm that the following items are present in the figure legend, table legend, main text, or Methods section.

n/a	Confirmed	
☐	☒	The exact sample size (*n*) for each experimental group/condition, given as a discrete number and unit of measurement
☐	☒	A statement on whether measurements were taken from distinct samples or whether the same sample was measured repeatedly
☐	☒	The statistical test(s) used AND whether they are one- or two-sided<br>*Only common tests should be described solely by name; describe more complex techniques in the Methods section.*
☒	☐	A description of all covariates tested
☐	☒	A description of any assumptions or corrections, such as tests of normality and adjustment for multiple comparisons
☐	☒	A full description of the statistical parameters including central tendency (e.g. means) or other basic estimates (e.g. regression coefficient) AND variation (e.g. standard deviation) or associated estimates of uncertainty (e.g. confidence intervals)
☐	☒	For null hypothesis testing, the test statistic (e.g. *F*, *t*, *r*) with confidence intervals, effect sizes, degrees of freedom and *P* value noted<br>*Give P values as exact values whenever suitable.*
☒	☐	For Bayesian analysis, information on the choice of priors and Markov chain Monte Carlo settings
☒	☐	For hierarchical and complex designs, identification of the appropriate level for tests and full reporting of outcomes
☒	☐	Estimates of effect sizes (e.g. Cohen's *d*, Pearson's *r*), indicating how they were calculated

Our web collection on statistics for biologists contains articles on many of the points above.

Software and code

Policy information about availability of computer code

Data collection	*Provide a description of all commercial, open source and custom code used to collect the data in this study, specifying the version used OR state that no software was used.*
Data analysis	CRISPR/Cas9 screen data analysis: MAGeCK 0.5.6 (https://sourceforge.net/p/mageck/wiki/Home/);<br>Data visualization and stat:GraphPad PRISM 7; Microsoft Excel 2016; Cytoscape software with ClueGo plug-in

For manuscripts utilizing custom algorithms or software that are central to the research but not yet described in published literature, software must be made available to editors/reviewers. We strongly encourage code deposition in a community repository (e.g. GitHub). See the Nature Research guidelines for submitting code & software for further information.

Data

Policy information about availability of data

All manuscripts must include a data availability statement. This statement should provide the following information, where applicable:

- Accession codes, unique identifiers, or web links for publicly available datasets
- A list of figures that have associated raw data
- A description of any restrictions on data availability

The results of CRISPR screens are included in the manuscript as Supplementary Data 1 & 2. All other datasets generated during the study are available from the corresponding author upon reasonable request.

Field-specific reporting

Please select the one below that is the best fit for your research. If you are not sure, read the appropriate sections before making your selection.

☒ Life sciences ☐ Behavioural & social sciences ☐ Ecological, evolutionary & environmental sciences

For a reference copy of the document with all sections, see nature.com/documents/nr-reporting-summary-flat.pdf

Life sciences study design

All studies must disclose on these points even when the disclosure is negative.

Sample size	No statistical methods were used to pre-determine sample size. Sample sizes (at least 3 independent experimental replicates in most experiments, unless indicated otherwise) were chosen based on the standard practices of the field.
Data exclusions	No data were excluded from the analysis.
Replication	All attempts at replication were successful.
Randomization	For in vivo studies, animals were randomised at day 0, prior to treatment. Samples used in cell biology experiments were not randomised.
Blinding	The investigator performing pain measurements in in vivo experiments was blinded to the experimental design/identity of injection. In cell biology experiments the investigators were not blinded during data collection and analysis.

Reporting for specific materials, systems and methods

We require information from authors about some types of materials, experimental systems and methods used in many studies. Here, indicate whether each material, system or method listed is relevant to your study. If you are not sure if a list item applies to your research, read the appropriate section before selecting a response.

Materials & experimental systems

n/a	Involved in the study	
☐	☒	Antibodies
☐	☒	Eukaryotic cell lines
☒	☐	Palaeontology
☐	☒	Animals and other organisms
☒	☐	Human research participants
☒	☐	Clinical data

Methods

n/a	Involved in the study	
☒	☐	ChIP-seq
☒	☐	Flow cytometry
☒	☐	MRI-based neuroimaging

Antibodies

Antibodies used	ATP2B1 antibody (#ab3528) was purchased from Abcam. SGMS1 antibodies (#ABC732) were purchased from Merck Millipore. Beta-actin antibody (#4970) was purchased from Cell Signaling Technology, Inc. Horseradish peroxidase-conjugated secondary antibodies (#31460) was purchased from Thermo Fisher Scientific
Validation	Antibodies were validated using knockout cell lines.

Eukaryotic cell lines

Policy information about cell lines

Cell line source(s)	293LTV cells: Cell Biolabs<br>HeLa: a gift from Dr. Adam R. Cole, Garvan Institute<br>HAP1: a kind gift from Thijn R. Brummelkamp (Netherlands Cancer Institute) (PMID: 21866103)
Authentication	HeLa cell lines were authenticated using STR DNA profiling. HAP1 and 293LTV were not formally authenticated.
Mycoplasma contamination	All cell lines tested negative for mycoplasma.
Commonly misidentified lines (See ICLAC register)	No commonly misidentified cell lines were used in the study.

Animals and other organisms

Policy information about studies involving animals; ARRIVE guidelines recommended for reporting animal research

Laboratory animals	All mice in this study were male FVB/NJ mice aged 10-15 weeks obtained from the Animal Resource Centre, WA, Australia.
Wild animals	The study did not involve wild animals
Field-collected samples	The study did not involve samples collected from the field.
Ethics oversight	All experiments were approved by the Animal Ethics Committee at the University of Sydney under protocol 1196.

Note that full information on the approval of the study protocol must also be provided in the manuscript.
